# Evolutionary optimization for risk-aware heterogeneous multi-agent path planning in uncertain environments

**DOI:** 10.3389/frobt.2024.1375393

**Published:** 2024-08-13

**Authors:** Fatemeh Rekabi Bana, Tomáš Krajník, Farshad Arvin

**Affiliations:** ^1^ Swarm and Computational Intelligence Laboratory (SwaCIL), Department of Computer Science, Durham University, Durham, United Kingdom; ^2^ Artificial Intelligence Center, Faculty of Electrical Engineering, Czech Technical University in Prague, Prague, Czechia

**Keywords:** multi-agent, path planning, probabilistic roadmap, collision avoidance, genetic optimization, bio-hybrid systems

## Abstract

Cooperative multi-agent systems make it possible to employ miniature robots in order to perform different experiments for data collection in wide open areas to physical interactions with test subjects in confined environments such as a hive. This paper proposes a new multi-agent path-planning approach to determine a set of trajectories where the agents do not collide with each other or any obstacle. The proposed algorithm leverages a risk-aware probabilistic roadmap algorithm to generate a map, employs node classification to delineate exploration regions, and incorporates a customized genetic framework to address the combinatorial optimization, with the ultimate goal of computing safe trajectories for the team. Furthermore, the proposed planning algorithm makes the agents explore all subdomains in the workspace together as a formation to allow the team to perform different tasks or collect multiple datasets for reliable localization or hazard detection. The objective function for minimization includes two major parts, the traveling distance of all the agents in the entire mission and the probability of collisions between the agents or agents with obstacles. A sampling method is used to determine the objective function considering the agents’ dynamic behavior influenced by environmental disturbances and uncertainties. The algorithm’s performance is evaluated for different group sizes by using a simulation environment, and two different benchmark scenarios are introduced to compare the exploration behavior. The proposed optimization method establishes stable and convergent properties regardless of the group size.

## 1 Introduction

Exploring animal–robot interactions involves the ambitious goal of comprehending how living species behave in the presence of robotic systems. Driven by a range of motivations, biologists and robotic researchers are compelled to investigate these hybrid dynamics, recognizing their potential to revolutionize biological–artificial integration (Romano et al., 2019). For instance, let us consider the concept of ecosystem hacking through a multi-robot system designed to influence honeybee colonies by interacting with the queen (Stefanec et al., 2022). Another example involves the utilization of a robotic hive to investigate collective behavior within this hybrid context concerning Western honeybees (Barmak et al., 2023). However, transition from the concept to real-world scenarios demands more sophisticated algorithm development, particularly for multi-agent systems that consider cooperation between the robots. For instance, the multi-arm robot presented in Rekabi-Bana et al. (2023), which is responsible for managing various tasks for the biological experiments described in Stefanec et al. (2022), requires an elaborate framework for decision-making, path planning, and motion control to achieve the expected goals. Although investigating the hybrid collaborative systems’ behavior results in extensive challenges for algorithm design, the former applications that utilized multi-agent solution challenges (Zlot et al., 2002; Amigoni et al., 2017; Vinyals et al., 2019) can be inspiring to design new frameworks compatible with new problems. Those algorithms allow the formulation of diverse cooperative frameworks and evaluate various aspects of multi-agent systems to solve the complexities. In addition, utilization of a team of heterogeneous robots with different capabilities creates a more versatile system that manages different sensing and actuation tasks, although it will increase the control complications (Rizk et al., 2019). In similar multi-robot applications such as exploration missions in disaster relief or search and rescue, cooperating robots spread throughout the targeted area to obtain its map as quickly as possible, while satisfying various constraints, such as communication (Rouček et al., 2020) or physical couplings between robots (Rekabi-Bana et al., 2023). These scenarios often require the robots to operate in adverse conditions, such as rain and wind, and they also experience conditions of reduced visibility due to smoke, fire, or dust (Tranzatto et al., 2022). Furthermore, in cases such as pollution source localization (Štibinger et al., 2020) or underwater inspection (Xiang et al., 2010), the robotic team has to assume or keep a specific formation to exploit all agents’ sensing capabilities. These scenarios require that the exploration methods take into account the environmental disturbances and uncertainties. Such scenarios have many features in common with the scenarios where robots should work in environments such as a hive, which is a confined and adverse environment. This paper presents a risk-aware path-planning algorithm that allows a group of robots to find safe trajectories, while considering the formation objectives for data collection in an adverse environment where actuation disturbances and sensor uncertainties affect the performance considerably.

### 1.1 Background

Various missions and objectives employ different approaches to describe the multi-agent system management problem (Dorri et al., 2018). For instance, Markov decision process (Liu et al., 2020); MAPF problem formulation, which is a layered optimization framework (Stern et al., 2019); and sampling-based approaches such as Monte Carlo simulation (Dalmasso et al., 2021) are some of the approaches that can describe the uncertainties in the environment properly and result in more robust solutions.

Although problem formulation is a key element to reach the main objectives, different methods might be applicable to solve those problems that become impossible to solve analytically in most cases according to the complexity order. Regardless of the problem formulation, two prevalent attitudes toward finding solutions are reinforcement learning (Qie et al., 2019; Semnani et al., 2020) and evolutionary optimization (Biswas et al., 2017; Li et al., 2021; Bahaidarah et al., 2024). Although the structures of both approaches are quite similar, various studies demonstrate differences between those methods to establish the advantages and weaknesses of both in different applications (Di Mario et al., 2013; Das et al., 2016). One of the best descriptions for the multi-robot path-planning problem is that it exploits combinatorial optimization to convert the discretized states into an efficient framework for optimization (Yu and LaValle, 2013; Okubo and Takahashi, 2023). Although the combinatorial problems allow considering multiple states and effective features regarding the mission objectives, the optimization search space becomes highly complicated and nonlinear. Some studies demonstrate that reinforcement learning is capable of solving such problems (Bengio et al., 2021). However, evolutionary optimization and particularly the genetic algorithm are a dominant methods for solving such problems according to their reliable heuristic behavior and convergence capability (Blum et al., 2011; Juan et al., 2015). Therefore, if a well-defined combinatorial optimization is used to describe the multi-agent path planning, the genetic algorithm will be a considerable approach for finding the solution. Although some research studies developed promising frameworks employing customized forms of the genetic algorithm to solve the pathfinding problem in 2D workspaces (Kala, 2012; Nazarahari et al., 2019) or independent coverage for large areas (Sun et al., 2019), many robotic applications in real-world scenarios demand considering other aspects such as uncertainties, robustness, and cooperative performance to fulfill their objectives. Therefore, the development of new optimization frameworks to investigate other practical objectives facilitates the applicability of employing reliable multi-agent robotic systems in real-world scenarios.

Path planning in an uncertain environment, which increases the risk of collision with the static or dynamic obstacle, is another challenging aspect that has been investigated in many recent studies (Rafai et al., 2022). One of the promising solutions to confront the uncertainties, particularly with the environment, is the risk-aware framework to avoid collisions with the obstacles (Pereira et al., 2013; Cai et al., 2021). However, such risk-aware frameworks can consider various types of risks in dynamic environments to address not only the collision but also other risk factors that cause mission failure (Barbosa et al., 2021).

On the other hand, the basic framework for pathfinding has a significant influence on the system’s performance. Although many different approaches are studied for path planning in various applications (Galceran and Carreras, 2013), the probabilistic roadmap (PRM) algorithm (Kavraki et al., 1996) proposed a flexible foundation that is adaptable to a wide range of planning problem formulation and optimization techniques to make robust planning frameworks, particularly in uncertain environments (Agha-mohammadi et al., 2014; Ravankar et al., 2020).

### 1.2 Contributions

This paper proposes a novel form of genetic optimization to find a solution for a multi-agent robotic system employing a risk-aware PRM scheme that was presented in Rekabi-Bana et al. (2024) as a robust framework for one robot to explore an area efficiently with minimum failure risk. The proposed planning algorithm utilizes the genetic algorithm in conjunction with the single-agent exploration method to find a conflict-free solution that enforces the agents exploring all subdomains together. Each subdomain is considered a district in the exploration area that includes at least one exploration point. Moreover, it considers the risk of collision with the obstacles according to a risk assessment function obtained from *a priori* knowledge of the environment and the robot’s dynamics. However, according to the problem definition, the risk assessment function can be defined to consider different sources of failure risk according to different scenarios. The proposed optimization framework allows incorporating the risk of failure according to the environmental conditions and, therefore, results in a reliable solution for exploration scenarios in workspaces with uncertainties. It allows the team to combine the data collected from each district to obtain as much information about the explored environment as possible. Therefore, the mission planning will include finding a proper formation shape for data collection, allocating the destinations to the agents, and collision-free pathfinding through the environment considering obstacle avoidance. The algorithm is capable of path planning in both 2D and 3D environments. Therefore, it will be applicable to use in the robotic system introduced in Rekabi-Bana et al. (2023) and small drones as well. Furthermore, the secondary objective is to develop a compatible path-planning algorithm with the distributed control framework designed for a multi-drone system (Rekabi-Bana et al., 2021) to make an entire autonomous robust framework for a group of flying robots to explore uncertain environments. The optimization scheme considers the traveling distance and the failure probability to determine the cost function. According to the problem definition, the exploration subdomain sequence, the agents’ destination in each district, and the priority order to find a collision-free set of trajectories are considered effective parameters that should be optimized to reach the mission objective. The combinatorial nature of the problem implies a particular structure to efficiently find the optimum point. Therefore, a customized structure is introduced to generate the chromosomes, reproduce the population, and analyze the convergence in each generation. The algorithm was designed as a centralized motion planning framework and has no dependencies on the agents’ communication. Therefore, this algorithm is applicable to cases where the robots are working close to the ground station and have a reliable network with it. The main contributions of this paper can be summarized as follows:

• Development of a multi-agent collision-free PRM-based path-planning algorithm for a group of robots working in an arbitrary 2D or 3D environment to explore the area cooperatively. The proposed algorithm allows the team to explore the area in a particular formation geometry for application in the multi-arm manipulator described in Rekabi-Bana et al., (2023).

• Proposing a new customized genetic framework to solve the path-planning problem as a combinatorial optimization. The proposed structure establishes a stable converging performance regardless of the number of agents in the multi-agent system.

The subsequent sections of the paper are structured as follows: Section 2 outlines the problem formulation, followed by Section 3 presenting the genetic algorithm framework developed to address the depicted problem. Section 4 explains the simulation environment and the evaluation procedure, and Section 5 demonstrates the results derived from the analysis. Finally, Section 6 provides the concluding remarks for the paper.

## 2 Problem formulation

The first step toward the algorithm design is describing the problem mathematically to formulate the objectives and constraints. This paper aims to develop a new framework for heterogeneous multi-agent path planning for a team of robots with different sensing capabilities exploring an uncertain environment. The robots should stay in a particular formation shape to maintain reliable communication, exploiting the measurement and actuation capabilities together, and comply with coupling constraints according to the robots’ dependencies on each other. The collision-free multi-agent path planning for area exploration is described and formulated in this section. The exploration area, $A_{e}\subset R^{3}$ , is described as an arbitrary three-dimensional space according to the following definition:

$$\begin{array}{ll} A_{e} & =F\cup O\\ A_{e} & =\left\{{\left[\begin{array}{ccc} x_{e} & y_{e} & z_{e} \end{array}\right]}^{T}|x_{e}^{\min}\leq x_{e}\leq x_{e}^{\max},\right.\\ & \;\left.y_{e}^{\min}\leq y_{e}\leq y_{e}^{\max},z_{e}^{\min}\leq z_{e}\leq z_{e}^{\max}\right\}, \end{array}$$ (1)

where $x_{e}^{\min},\ldots ,z_{e}^{\max}$ represents the workspace borders and O and F demonstrate the occupied and free space, respectively.

It is assumed that the workspace for the exploration is mapped using the risk-aware PRM method and according to the available data set from the environment, including the workspace borders and occupied areas’ boundaries. Nevertheless, the proposed algorithm in Rekabi-Bana et al. (2024) needs few data, considers uncertainties of the environment data, and creates a pathfinding graph according to a predefined admissible risk to handle the collision risk with obstacles. Therefore, the environment graph is described as follows:

$$\begin{array}{ll} G_{e} & =\left(V_{e},E_{e}\right)\\ V_{e} & =\left\{v_{k}^{e}\in A_{e}\right\},\;k=1,\ldots ,N_{\mathrm{prm}}^{\mathrm{node}},R\left(v_{k}^{e}\right)\leq R_{ad}\\ E_{e} & =\left\{e_{t}^{e}=\left(v_{i}^{e},v_{j}^{e}\right)\right\},\;t=1,\ldots ,N_{\mathrm{prm}}^{\mathrm{edge}},R\left(e_{t}^{e}\right)\leq R_{ad}, \end{array}$$ (2)

where $G_{e}$ is the environment exploration graph; $v_{k}^{e}\in R^{3}$ and $e_{t}^{e}\in R^{3\times 2}$ represent the graph nodes and graph edges, respectively; $N_{\mathrm{prm}}^{\mathrm{node}}$ and $N_{\mathrm{prm}}^{\mathrm{edge}}$ are the number of nodes and edges in the graph, respectively; $R_{ad}$ is the admissible collision risk considered to calculate the PRM nodes and edges, and R(.) is the risk assessment function obtained according to Rekabi-Bana et al. (2024). The exploration objective is to cover a set of randomly distributed exploration points in the workspace to harvest information. The workspace is divided into districts according to the robots’ sensors’ capabilities for coverage. Each district includes some of the exploration points, and the robots should cover each of them cooperatively. According to the number of agents, the agents’ sensing radius, and the exploration targets’ distribution, the destination points form an equilateral polygon to place the agents in a formation shape that allows the agents to cover the exploration points as much as possible. Consequently, the formation shape at each district is defined according to the following equation:

$$\begin{array}{ll} C_{fr}^{d} & ={\bar{P}}_{ex}^{d}\\ R_{fr}^{d} & =F^{R}\left(\|\sigma \left(P_{ex}^{d}\right)\|,{\bar{R}}^{s}\right)\\ \alpha_{fr}^{d} & =∠\sigma \left(P_{ex}^{d}\right),\;d=1,\ldots ,N_{\mathrm{dist}}, \end{array}$$ (3)

where $P_{ex}^{d}=\left\{p_{i_{ex}}^{d}\in R^{3}\right\}$ is the set of exploration target points, $C_{fr}^{d}$ is the center point according to $P_{ex}^{d}$ , $R_{fr}^{d}$ is the formation radius, $\alpha_{fr}^{d}$ is the formation polygon rotation angle, $R^{s}=\left\{r_{i}^{s}\in R\right\}$ is the set of sensing radii according to the agents’ measurement system capability, and $N_{\mathrm{dist}}$ is the number of districts for exploration. In addition, $\bar{\left(.\right)}$ is the mean value, ‖(.)‖ is the norm, ∠(.) is the vector angle, and σ(.) is the variance operator. $F^{R}$ is a function used to determine the circumradius of the polygon and is defined as follows:

$$\begin{array}{ll} & F^{R}\left(\|\sigma \left(P_{ex}^{d}\right)\|,{\bar{R}}^{s}\right)=\left\{\begin{array}{c} \|\sigma \left(P_{ex}^{d}\right)\|,\;\;if\;\;\|\sigma \left(P_{ex}^{d}\right)\|\leq {\bar{R}}^{s}\;\\ \frac{{\bar{R}}^{s}+\|\sigma \left(P_{ex}^{d}\right)\|}{2},\;\;if\;\;\|\sigma \left(P_{ex}^{d}\right)\|\geq {\bar{R}}^{s}.\; \end{array}\right. \end{array}$$ (4)

**FIGURE 1:**
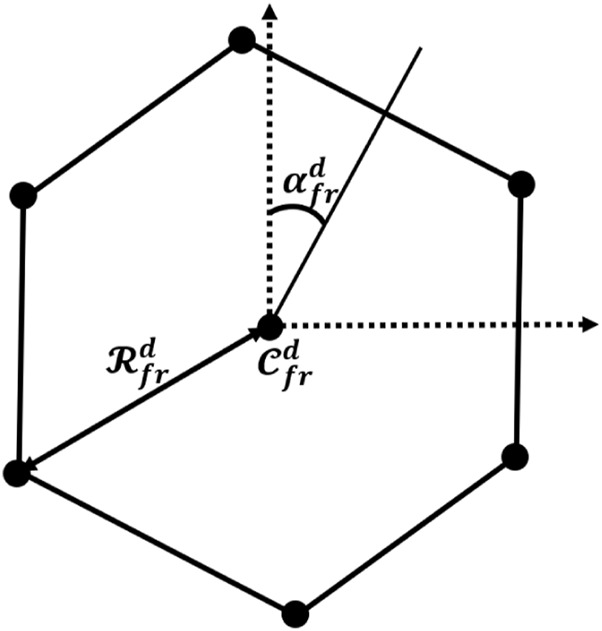
Formation shape for six agents according to the definitions in Equation 3.

The formation shape obtained from Equation 3 represents the robots’ target positions, allowing them to access the exploration points as much as possible for data collection using this configuration. Figure 1 presents the graphical perspective of Equation 3. According to the aforementioned environment partitioning and target formations, the path-planning algorithm should find a set of collision-free paths in the environment graph to pass the agents through the safe area and prevent them from colliding. Additionally, the traveling distance for the group should be minimized, and the agents should go through each district once. Furthermore, placing the agents in the formation considering their sensing radius is important to maximize the number of exploration targets. Therefore, path planning can be defined as a constrained optimization problem described in the following equation:

$$\begin{array}{ll} if\;\Pi & =\left\{\pi_{i}\right\},\pi_{i}=\left\{\pi_{i}^{d}\right\}\subset \Phi \left(G_{e}\right),\;i=1,\ldots ,N_{ag}\\ \Pi^{*} & =\mathrm{arg min}\left(J_{\mathrm{mission}}\right),\\ & \;s.t.\;\pi_{i}\cap \pi_{j}=Ø\;\;if\;\;i\neq j,\;\pi_{i}\cap O=Ø\\ & \;d=1,\ldots ,N_{\mathrm{dist}}, \end{array}$$ (5)

considering

$$J_{\mathrm{mission}}=L\left(\Pi \right)+\Sigma_{d=1}^{N_{\mathrm{dist}}}\Sigma_{i_{ex}=1}^{N_{ex}^{d}}\lambda_{i_{ex}}^{d}S\left(p_{i_{ex}}^{d}\right),$$ (6)

$$L\left(\Pi \right)=\gamma P\left(\Pi \right)+\Sigma_{d=1}^{N_{\mathrm{dist}}}\Sigma_{i=1}^{N_{ag}}D\left(\pi_{i}^{d}\right),$$

$$S\left(p_{i_{ex}}^{d}\right)=\left\{\begin{array}{c} 0\;\;if\;\;min\left(\left\|p_{i_{ex}}^{d}-q_{i}^{d}\right\|\right)\leq r_{i}^{s}\;\\ 1\;\;otherwise\; \end{array}\right.,\;\;i=1,\ldots ,N_{ag},$$

where $\pi_{i}$ is the path for the $i_{th}$ agent, $\pi_{i}^{d}$ is part of $\pi_{i}$ that allows the $i_{th}$ agent to reach its destination in $d_{th}$ districts, $\Phi \left(G_{e}\right)$ is the set of all possible paths in the environment graph, $J_{\mathrm{mission}}$ is the mission overall cost, $D\left(\pi_{i}^{d}\right)$ is the distance traveled by the $i_{th}$ agent through $\pi_{i}^{d}$ , $P\left(\pi_{i}\right)$ is the failure probability for Π implementation regarding the collision between the agents or with obstacles, γ is the importance factor for the failure probability, $q_{i}^{d}$ is the destination point for the $i_{th}$ agent in the $d_{th}$ district, and $\lambda_{i_{ex}^{d}}$ is the importance weight for each $p_{i_{ex}}^{d}$ exploration point. According to Equations 5, 6, the solution of the problem includes a set of paths that belong to the possible paths in the environment graph which do not undergo any collision with each other. On the other hand, the agents should reach a target formation in each district, and therefore, they should use paths that might be close to each other. Consequently, making a set of collision-free paths needs to prevent use of the same nodes and edges for two agents simultaneously. Therefore, in this paper, the proposed algorithm for path planning considers three main parameters that affect the mission cost:

• The order of districts that the agents should pass through to explore the area.

• The destination point of each agent in the formation.

• The order of the path association in the exploration graph for the agents.

The procedure to solve the problem, defined in Equations 5, 6, using genetic optimization is described in the next section.

## 3 GA multi-agent path planning

The path-planning algorithm is a combinatorial optimization problem. The final set of paths includes two main features: 1) the best combination of the districts that the agents should pass through and 2) the best mapping from the agents’ IDs and the destination points in the target formation. Furthermore, the paths should have no similar nodes and edges so as to reduce the collision risk between the agents. In addition, it is necessary to check if the final trajectories through all the waypoints are collision-free. The proposed strategy to find collision-free paths in the graph is explained in Algorithm 1. Therefore, those features should be considered in the genetic algorithm to find the solution.

### 3.1 Genetic population

**FIGURE 2:**
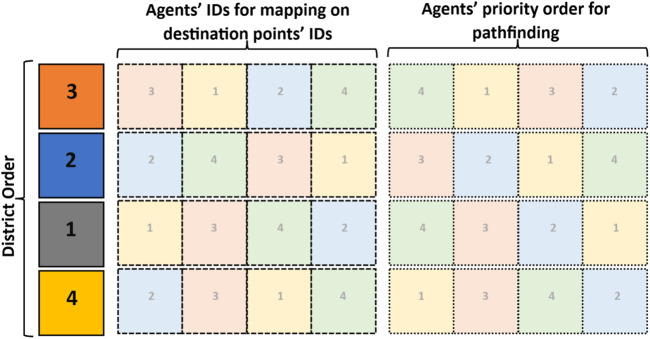
A sample chromosome (for four exploration subdomains and four agents) to demonstrate the structure defined to solve the collision-free multi-agent pathfinding.

According to the features defined for the final solution, the suggested chromosome structure is composed of the district identifiers, the agents’ identifiers allocated to the destination points, and the order of agents’ identifiers for collision-free pathfinding. Therefore, each chromosome can be described as a matrix that is structured as shown in Figure 2.

Algorithm 1 Collision-free pathfinding.

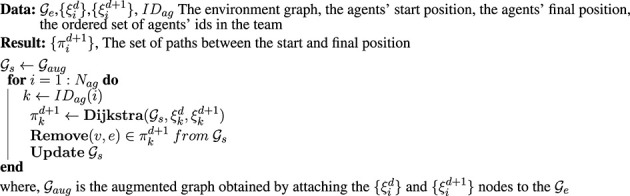

According to the proposed structure, the initial population is created by generating $N_{\mathrm{pop}}$ random permutations for the districts’ sequence, agents’ identifiers for mapping to the destination points, and the sequence for pathfinding in the graph. The population size is determined according to Ahn and Ramakrishna (2002) to comply with the nature of the problem.

### 3.2 Evolutionary regeneration

The first reproduction method is to transfer the $N_{el}$ best chromosomes directly to the next generation. However, the proposed 2D chromosome structure and the combinatorial nature of the problem do not allow for using popular strategies for regeneration through crossover and mutation. Therefore, two new strategies are proposed and applied to create the next generation from the current parents. The first strategy is a one-parent crossover according to the following steps:

• Selection of $N_{\mathrm{crs}}$ random chromosomes from the current population as the crossover parents.

• For each selected chromosome, one or two random breakpoints for the first column and a permutation are determined to reorder the districts’ sequence.

• For each selected chromosome, two or three random columns will be selected among the next $N_{ag}$ columns (depending on the number of agents), and their position will be switched according to a random permutation to rearrange the agents’ destination points in the target formations.

• For each selected chromosome, two random columns are selected among the last $N_{ag}$ columns, and their position will be switched to reorder the agents’ priority for pathfinding in the graph.

Considering the above procedure, each child will inherit some of the parents’ characteristics and shows new behavior. Furthermore, the parents are selected randomly regardless of their achieved cost function in the current generation to prevent the algorithm from following the current best population members and preserve the algorithm’s heuristic performance. In addition, a proposed mutation is used to generate $N_{\mathrm{mut}}=N_{\mathrm{pop}}-\left(N_{el}+N_{\mathrm{crs}}\right)$ remaining children to complete the next-generation chromosomes. The proposed mutation works as follows:

• For each selected chromosome, a random number between 1 and 3 is generated to determine which part of the chromosome should be mutated.

• If the first part is selected, then the current district sequence will be replaced by a random permutation for the districts’ identifier to generate a completely different one.

• If the second part is chosen, then a random row will be designated and the mapping order will be replaced with a random permutation of the agents’ identifiers.

• If the third part is chosen, a random row will be selected and the pathfinding priority order will be replaced with a random permutation of the agents’ identifiers.

The further important parameters significantly affecting the algorithm’s behavior are the number of chromosomes that will be selected to pass through the elite, crossover, or mutation process for regeneration. In this paper, the following process is proposed to determine those parameters:

• $N_{el}$ is a constant value, and it is equivalent to the top 5% of the population.

• $N_{\mathrm{crs}}$ is variable to cover between 80% and 90% of the population. Its value depends on the population density. The higher the population density, the lower the $N_{\mathrm{crs}}$ to allow mutation to reinforce the heuristic features.

• The number of chromosomes for the mutation will be obtained from $N_{\mathrm{mut}}=N_{\mathrm{pop}}-\left(N_{el}+N_{\mathrm{crs}}\right)$ to keep the population size over generations.

### 3.3 Convergence criteria

There is no theoretical method to establish the final convergence for the genetic algorithm in an arbitrary problem. Therefore, it is necessary to stop the algorithm while it satisfies a set of predefined conditions. Different types of convergence analysis are applicable to the genetic algorithms in different problems (Safe et al., 2004). In this paper, the following criteria are considered to determine the algorithm’s convergence:

• Elite group sustainability: if the chromosomes that reached the top 5% remain in their positions for at least 20 generations, it means the search is converged, and it is more likely for the algorithm to stay on the same point as the optimum solution in the next generations.

• The cost function gradient: according to the combinatorial nature of the problem, it is possible to have more than one solution as the best combination of features to satisfy the minimum value for the cost function. Therefore, it is necessary to consider another condition rather than an elite set of chromosome sustainability. The cost function gradient is another condition that demonstrates the algorithm’s convergence. In this paper, if the cost function gradient remains less than 2% for 50 generations, it means the algorithm has reached its final convergence.

• Population distribution: checking the population distribution is not very easy in combinatorial problems, particularly with the 2D chromosome structure. Accordingly, a new strategy is proposed to compare the current population with the previous generations to check how many new points in the search domain are considered in the current generation. For this reason, a random key is generated in the beginning to select particular genes from the chromosomes and devise a comparison feature to evaluate the heuristic performance level. Accordingly, if the number of new chromosomes remains less than 2% of the population size for more than 20 generations, it means the algorithm has reached its final convergence.

• The maximum iteration: a maximum number of iterations equivalent to 10 times the population size is considered to prevent computational overloading.

## 4 Simulation environment and evaluation

The performance evaluation for the proposed algorithm is accomplished using a simulation environment presented in our previous works (Rekabi-Bana et al., 2020; 2021). Furthermore, to guarantee cooperative stability and robustness against uncertainties and disturbances, the distributed $NLH_{\infty }$ presented in Rekabi-Bana et al. (2021) is applied. The test scenario includes an arbitrary workspace with three obstacles. The procedure to map the environment and create a risk-aware exploration graph is similar to that mentioned in our previous work, which is discussed in Rekabi-Bana et al. (2024).

Furthermore, in order to compare the algorithm’s behavior for different group sizes, four conditions are studied. The group size varies from three to six agents. The population size for the optimization is determined as 20 times the number of agents. That value is obtained by a trade-off between the computational burden and the heuristic performance of the algorithm. In each iteration, a set of 15 simulations is implemented considering the robots’ dynamics and environmental disturbances to evaluate the traveling distance and failure probability due to collision between the agents or the obstacles. Furthermore, the number of exploration points covered according to the agents’ sensing radius and their destination position in the formation is determined to calculate the second part of the cost function. Accordingly, the cost function will be calculated for each chromosome based on those values afterward.

In order to compare the proposed algorithm’s performance with that of other planning schemes, two other methods are considered. It is assumed that there is no possibility of collision with the obstacles, and the agents can fly between different formations directly in both comparison algorithms. In the first scenario, an ideal exploration is considered, which includes the shortest path between all the destination points. In the second case, the random search is considered for the group, which is a classic method for exploration. The coverage score is defined to consider the number of exploration points in each district proportionally and the points scattering diversely. It means that the districts with more exploration points which are less sparse have the highest score. It is evident that if the agents cover all the districts, they will achieve the highest score.

**FIGURE 3:**
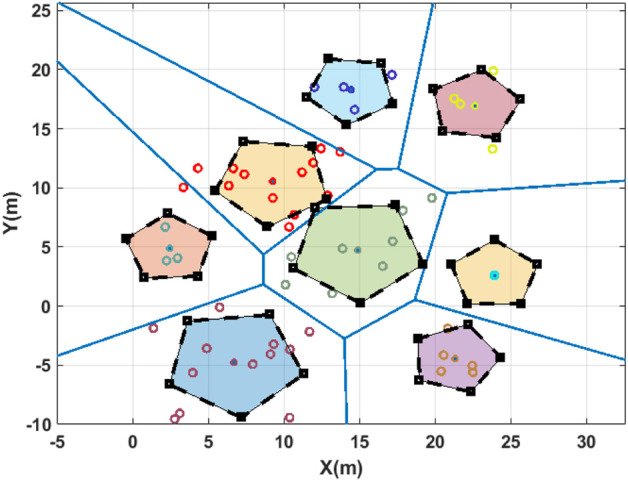
2D representation of a mission for a group of five robots in an arbitrary workspace. Blue lines show the borders for the subdomains, circular points demonstrate the exploration points, and square points and dashed lines depict the formation shape boundaries and the destination points in each district, respectively, which are represented by different colors.

Figure 3 demonstrates one of the sample missions for performance evaluation. It depicts how the exploration subdomains are formed and how the formation shape will look according to the exploration points’ distribution in each subdomain.

## 5 Results and discussion

The results obtained from the proposed algorithm implementation in the simulation environment are discussed in this section. According to the problem’s definition in Section 2, the preliminary stage before the optimization involves determining the target formations in each district to place the agents in the vicinity of exploration points for data collection. A sample for exploration points’ distribution, workspace partitioning, and target formations is presented in Figure 3.

### 5.1 Exploration with aerial robots

As is presented in Figure 1, the formation shape is considered an equilateral polygon regarding the number of agents in the exploration team. The size and orientation of the polygons are determined according to the exploration points’ distribution in each district and the data collection radius for the agents. Therefore, the more sporadic the exploration points, the larger polygon will be considered to place the agents in positions for a complete coverage.

**FIGURE 4:**
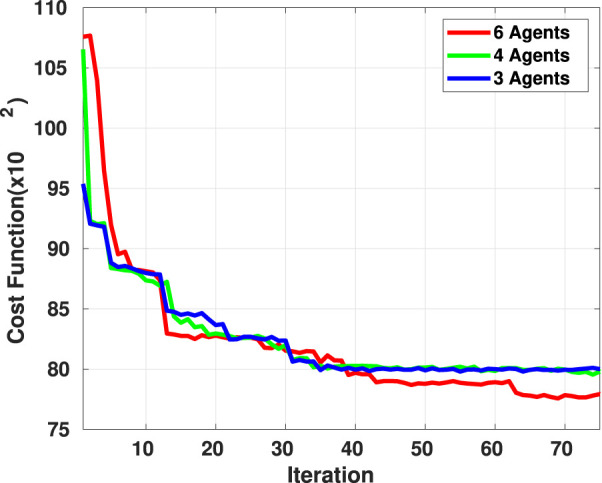
Best value of the cost function in the genetic optimization obtained for three, four, and six agents in the exploration team.

**FIGURE 5:**
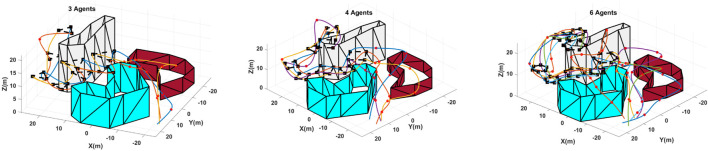
Path-planning results obtained from the proposed genetic optimization framework for three, four, and six agents in an arbitrary workspace. The obstacles’ boundaries are shown by gray, brown, and blue surfaces; the target formations are depicted by square points and dashed lines; the path waypoints are presented by red dots; and the final trajectories are demonstrated by colored lines.

The results obtained for the cost function’s best value at each generation are presented in Figure 4. The results demonstrated in Figure 4 show the first 75 iterations of the optimization for all the configurations. The result indicates that the algorithm structure is designed properly and the heuristic and optimization stability is not affected by the group size. However, the more agents in the group, the larger the optimization search domain, and accordingly, the more iteration is required to satisfy the convergence criteria. Furthermore, the simulation results obtained using the proposed planning algorithm for three different group sizes are presented in Figure 5, demonstrating the trajectories that each agent should pass to reach the allocated destination points in target formations.

**FIGURE 6:**
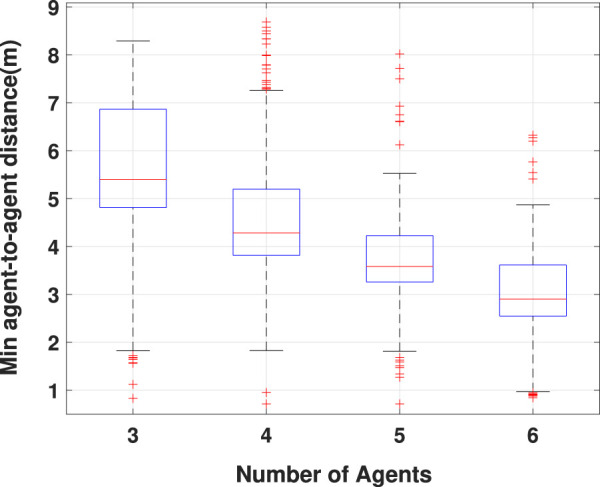
Collision-free behavior evaluation using the minimum agent-to-agent distance in four team configurations.

**FIGURE 7:**
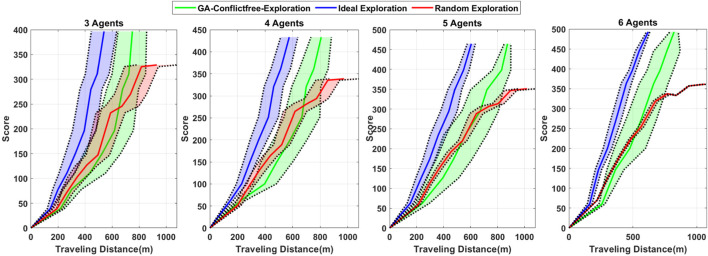
Comparison results for the proposed algorithm exploration performance in three group configurations. The solid lines represent the average performance, and the shaded area with dotted boundary lines demonstrates the 25% and 75% quantiles for the statistical results.

Although Figure 5 demonstrates the proposed algorithm’s output for path planning, it is necessary to analyze the minimum agent-to-agent distance criterion to evaluate the collision-free feature of the proposed multi-agent path-planning algorithm. The results obtained for that criterion in four team configurations are demonstrated in Figure 6. The results depicted in Figure 6 show that in all the studied conditions, the minimum agent-to-agent distance is more than almost 0.5 m, and no collision has occurred between the agents. Furthermore, it is evident that the variation bound of the distance between the agents decreases with increase in the number of agents. Although the presented results establish the capability of the proposed optimization for multi-agent collision-free path planning in an arbitrary environment, it is necessary to evaluate the proposed algorithm’s performance in comparison with other methods. The comparison results with two benchmark scenarios based on the statistical data obtained from multiple simulations for different configurations are presented in Figure 7. The first benchmark is the ideal exploration, which means that the agents explore the area by moving directly between each pair of districts regardless of the existing obstacles and collision risk according to the best combination of the districts obtained according to a TSP problem. The second benchmark is the random exploration without any risk of collision with the obstacle and directly between districts, as considered in the first benchmark. According to the results demonstrated in Figure 7, the behavior of the proposed algorithm is similar to the ideal benchmarks considered for each configuration, although it is supposed to avoid collision with obstacles and find the trajectory through the PRM graph. Furthermore, the proposed algorithm outperforms the random exploration by preventing the agents from repeatedly visiting the districts, which is a problem with the random exploration method.

Although the proposed algorithm provides exploration plans similar to an ideal case, the constrained optimization framework requires a considerable computation resource. Algorithm speed was analyzed using its implementation in MATLAB 2022b on a computer with a 12th Gen Intel(R) Core(TM) i9-1200k processing unit and 64 Gb of RAM. The results obtained for different numbers of agents, exploration districts, and environment dimensionality are presented in Table 1. It is clear that compared to the 3D environments, path planning in two-dimensional environments achieves relatively short run times, even with a significantly larger graph. The results also indicate that increasing the number of agents or districts increases the run time approximately linearly.

**TABLE 1:** Algorithm run-time in minutes for different scenarios in 3D and 2D environments with PRM graphs of 2,663 and 12,812 edges respectively.

	Number of districts			
	10	8	7	5
Three agents	15.34	12.22	10.68	7.63
Four agents	20.31	16.39	14.34	10.16
Five agents	25.53	20.36	17.83	15.14
Six agents	30.54	24.47	21.43	15.26

	Number of districts			
	10	7	6	5
Three agents	8.30	6.10	5.24	4.36
Four agents	12.72	7.95	6.86	5.72
Five agents	13.90	9.84	8.57	7.06
Six agents	16.62	11.76	10.09	8.45

3D: PRM graph with 2,663 edges.

2D: PRM graph with 12,812 edges.

### 5.2 Exploration of a honeybee colony with a multi-arm manipulator

**FIGURE 8:**
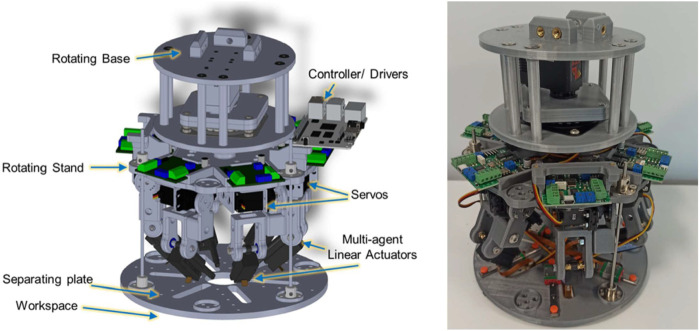
Multi-arm manipulator designed for data collection from the observation hive (Rekabi-Bana et al., 2023).

The proposed algorithm is also applied to determine a proper set of trajectories for the multi-arm robot designed for the RoboRoyale project (Rekabi-Bana et al., 2023). In this study, a set of robotic agents, attached to arms on the manipulator, will interact with the honeybee queen (Stefanec et al., 2022). Each agent will be equipped with a miniature sensor to retrieve information about the queen and the comb. The robot configuration is presented in Figure 8.

When the queen is active, the agents follow her and monitor her activity, and they detect where she lays the eggs. To assess the healthiness of the brood, the agents have to visit the locations of the previous egg-laying events and investigate the progress in the brood development. This requires that the agents leave the queen during the resting times and gather the observations of the brood as quickly as possible to minimize the risk of missing important queen activities. During the exploration, the agents have to avoid disturbing the colony and prevent collisions with any elements of the hive.

**FIGURE 9:**
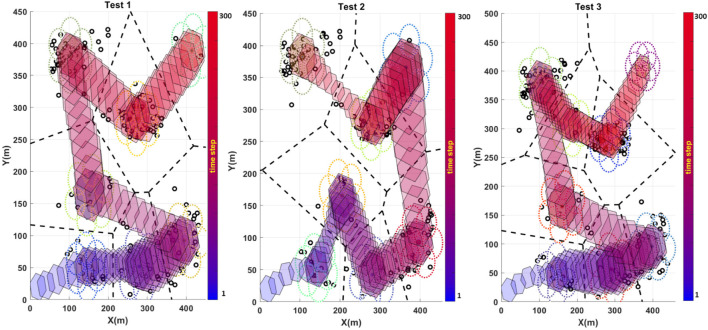
Results obtained for the multi-arm manipulator trajectory planning to cover egg-laying spots in three different sampling cases. The black circles represent the egg-laying spots, the black dashed lines represent the workspace partitioning according to the selected egg-laying samples, and the colored hexagon shows the footprint of the manipulator through time, starting from blue and shifting to red as time passes.

Our experiment is based on real egg-laying data collected with respect to a honeybee observation hive. There, an image processing system was used to determine a set of egg-laying events (Žampachů et al., 2022), and the events gathered over the 24-h period were considered target points for workspace partitioning and target formation determination. A geometrical constraint was also added to the optimization framework to comply with the robot’s coupling and kinematic constraints. Accordingly, the obtained results for three sets of egg-laying spots are demonstrated in Figure 9.

**FIGURE 10:**
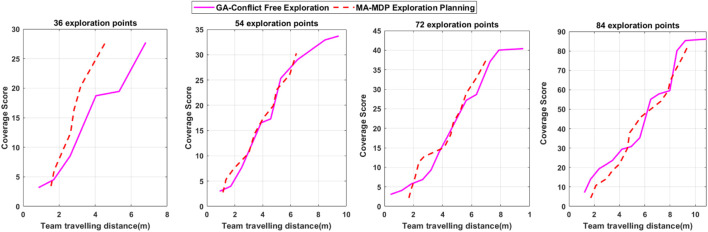
Comparison results in four scenarios with different numbers of exploration target points.

The presented results in Figure 9 demonstrate the optimization results and workspace partitioning in three cases. In each case, 150 egg-laying samples are selected randomly to make a different distribution. Therefore, the workspace partitioning and each district value for exploration would be different according to a new sample distribution. Accordingly, the resulting trajectory becomes different in each case and complies with the new sample distribution. The robot’s endpoint footprint presented in Figure 9 demonstrates how the algorithm attempts to find the best placement for the robot arms’ endpoint according to the kinematic constraints. The robot arms’ mechanical constraints make solid restrictions such as maximum and minimum diameter for expansion and retraction and the overall rotation, which do not allow for full coverage of target points in each domain. However, the proposed algorithm tries to attain the maximum exploration score by reaching the position to cover the maximum number of the target points at each district. The performance of our method is compared with that of an efficient algorithm recently proposed for a joint area coverage scenario with a team of robots (Nawaz and Ornik, 2023). The performance of both algorithms was evaluated in different conditions with different numbers of exploration target points sampled randomly from the egg-laying events. The results obtained from the comparison are shown in Figure 10. The results in Figure 10 show that the two algorithms achieve similar performance. However, our algorithm finds optimal efficient solutions considering additional criteria such as kinematic coupling and the conflict-free path between the agents, which are not considered in the MA-MDP framework proposed in Nawaz and Ornik (2023). Therefore, the comparison result establishes the efficiency and optimality of the algorithm as it complies with the practical requirements of the robots for the exploration.

### 5.3 Limitations and future works

According to the results presented in previous sections, it is evident that the proposed algorithm has some limitations, particularly regarding the run-time performance. The results demonstrated in Table 1 show that the current implementation framework is time-consuming and limited to applications that do not demand fast planning and re-planning. For instance, the studied case of a multi-arm manipulator for egg-laying coverage in an observation hive is one of the applications not needing prompt planning, and the proposed algorithm is a good solution for that application. However, implementing the algorithm with a more efficient programming system and employing parallel computation will enhance the performance and reduce the run-time to cover more practical robotic applications. Moreover, the centralized framework for the algorithm limited its applicability to those cases that rely on a ground station with a reliable communication link between the agents and the main processing unit. Therefore, developing a distributed form of the algorithm will be one of the future solutions that allow utilizing the proposed method for applications that have less reliance on the ground station.

## 6 Conclusion

This paper presents an innovative path-planning algorithm that leverages the risk-aware probabilistic roadmap (PRM) method, combined with a customized genetic optimization approach, which is compatible with the complexities of the path-planning problem. The algorithm prioritizes two key criteria: minimizing travel distance and reducing the probability of collision. These criteria collectively constitute the objective function guiding the optimization process. The proposed evolutionary framework employs a sampling-based method to compute the objective function. This method relies on a series of simulations that consider the dynamics of agents and environmental disturbances. Therefore, those simulation results can reflect the statistical characteristics of the objective function according to all random processes considered in the model. The optimization results demonstrate a reasonable convergence regardless of all the variations in the environment and team configuration. Furthermore, the algorithm’s effectiveness in ensuring collision-free trajectories is validated by an analysis of the minimum agent-to-agent distances along their paths. This analysis serves to underscore the collision-free properties inherent in the algorithm. A comparison of exploration scores for various group configurations demonstrates the superior performance of the proposed algorithm, positioning it as a promising optimization solution for benchmark cases. It outperforms random exploration methods. Importantly, while originally designed for finding three-dimensional trajectories for cooperative exploration executed by a group of drones, the algorithm possesses the adaptability needed to address the path-planning requirements of multi-arm robots operating in higher dimensions, while maintaining collision avoidance capabilities. Looking ahead, the current research envisions further enhancements, including real-world testing with physical robots and integration with distributed control algorithms, ultimately presenting a unified framework for multi-robot autonomous exploration in uncertain environments with dynamic disturbances. These future endeavors will contribute to a more comprehensive and practical solution for a wide range of robotic applications.

## Data Availability

The raw data supporting the conclusions of this article will be made available by the authors, without undue reservation. These will be available through the funding project website https://roboroyale.eu.
